# Identification of adenylyl cyclase isoforms mediating parathyroid hormone- and calcitonin-stimulated cyclic AMP accumulation in distal tubule cells

**DOI:** 10.1186/s12882-017-0712-5

**Published:** 2017-09-07

**Authors:** Wararat Kittikulsuth, Peter A. Friedman, Alfred van Hoek, Yang Gao, Donald E. Kohan

**Affiliations:** 1 Division of Nephrology, University of Utah Health Sciences Center, 1900 E 30 N, Salt Lake City, UT 84132 USA; 2 Department of Pharmacology and Chemical Biology, University of Pittsburgh, Pittsburgh, PA USA; 3 Department of Neurology, University of Utah Health Sciences Center, Salt Lake City, UT USA; 4 Salt Lake Veterans Affairs Medical Center, Salt Lake City, UT USA

**Keywords:** Distal convoluted tubule, Adenylyl cyclase, Calcitonin, Parathyroid hormone, Isoform

## Abstract

**Background:**

The distal convoluted tubule (DCT) is an important nephron site for parathyroid hormone (PTH) and calcitonin regulation of urinary divalent cation excretion. These hormones exert their effects on the DCT in substantial part through activation of adenylyl cyclase (AC); however, it is unknown which AC isoforms are involved.

**Methods:**

To examine this, two mouse DCT cell lines were studied: 209 and D1 cells. AC isoform mRNA expression was analyzed by real-time PCR. Cyclic AMP was measured using enzyme immunoassay.

**Results:**

Calcitonin, but not PTH, stimulated cAMP accumulation in 209 cells, while PTH, but not calcitonin, increased cAMP content in D1 cells. Both cell types expressed AC3, AC4, AC6, AC7, and AC9 mRNA; in both cell types, AC6 mRNA was most abundant, followed by AC9, then AC3 and AC7, with relatively very small amounts of AC4 mRNA. Microdissected mouse DCT had a similar pattern of AC isoform mRNA expression although AC5 mRNA was detected. Individual siRNA knockdown of AC6 and AC9 reduced calcitonin-stimulated cAMP accumulation in 209 cells and PTH-induced cAMP levels in D1 cells. Knockdown of AC3 had no effect on hormonal augmentation of cAMP in either cell line. Surprisingly, knockdown of AC7 increased calcitonin-induced cAMP accumulation in 209 cells as well as PTH-stimulated cAMP content in D1 cells.

**Conclusions:**

Taken together, these findings indicate that AC6 and AC9 mediate calcitonin- and PTH-stimulated cAMP accumulation in DCT cells, while activation of AC7 may paradoxically reduce the stimulatory effects of PTH and calcitonin on cultured DCT cAMP levels.

## Background

The distal convoluted tubule (DCT) is an important nephron site of electrolyte reabsorption, including Na^+^, Cl^−^, Ca^2+^ and Mg^2+^ [1]. Although the intracellular signaling pathways modifying DCT Na^+^/Cl^−^ cotransporter (NCC) activity have been the subject of intensive study, the regulation of DCT Ca^2+^ and Mg^2+^ transport is less well understood [1]. DCT transport of these divalent cations is controlled by multiple hormones; however, amongst these, parathyroid hormone (PTH) and calcitonin have emerged as being of particular importance [1]. While these hormones modulate multiple signaling systems within the DCT, a key initial step is activation of adenylyl cyclase (AC) to generate cAMP; PTH- and calcitonin-induced stimulation of cAMP increases DCT Ca^2+^ and Mg^2+^ reabsorption [1, 2]. Somewhat surprisingly, the characteristics of this initial AC activation by PTH and calcitonin are poorly understood. To our knowledge, no studies have examined which of the 9 membrane-bound AC isoforms are involved in PTH or calcitonin stimulated cAMP specifically in the DCT. In addition, to our knowledge, no studies have examined which AC isoforms mediate calcitonin-induced cAMP in any cell type. PTH-stimulated cAMP content has been reported to be mediated, at least in part, by AC6 in human embryonic kidney [3] and osteoblasts [4], while PTH-induced increases in endosomal cAMP content in osteosarcoma cells are partly mediated by AC2 [5]. Consequently, the present study was undertaken to define, for the first time, which AC isoforms mediate PTH and calcitonin increases in cAMP accumulation in the DCT using DCT cell lines as a model.

## Methods

### Animal study approval and animal handling

All experiments were carried out in accordance with and after approval by the University of Utah Health Sciences Center Institutional Animal Care and Use Committee. All mice were fed standard chow and water ad lib. No experimental procedures were performed on live animals. At the time of sacrifice for tissue harvest, mice were euthanized with enflurane and when breathing was stopped for 1 min, kidneys were harvested.

### Cell culture

Two mouse DCT cell lines, 209 and D1, were provided by Dr. Peter Friedman at the University of Pittsburgh. Both cell lines were initially derived from primary cultures of DCT cells that were simian virus transformed and cloned by limiting dilution, termed 209 cells [6]. The DCT phenotype has been confirmed by thiazide-inhibited Na^+^ and Cl^−^ uptake, thiazide-stimulated Ca^2+^ uptake, and absence of an effect of bumetanide (inhibits Na^+^/K^+^/2Cl^−^ transporter) [6]. The D1 cell line was derived from 209 cells stably transfected with the human PTH receptor and exhibits PTH-dependent cAMP accumulation [7]. Both cell lines were grown in 24-well plastic culture plates in 50:50 DMEM/F-12 (Gibco, Thermo Fisher Scientific, Waltham, MA) supplemented with 5% fetal bovine serum (FBS, Gibco) in a 5% CO_2_ incubator at 37 °C.

### siRNA studies

Both cell lines were grown to 50% confluence. Cells were treated for 24 h with 100 μl Opti-MEM Reduced Serum Medium (Life Technologies, Thermo Fisher Scientific) containing 1.5 μl Lipofectamine® RNAiMAX Transfection Reagent (Life Technologies) and 10 pmoles scrambled or AC isoform siRNA. Media was then removed and cells incubated with DMEM:F12 containing 1% FBS for 24 h (followed by mRNA analysis) or for 48 h (followed by cAMP and total protein determination). The siRNA (Origene, Rockville, MD) was: AC3 - SR422209A, AC6 - SR422280A-C, AC7 - SR422059A-C, AC9 - SR422722A-C, and scrambled controls.

### Cyclic AMP assay

Cells used for dose-response studies or in siRNA studies were treated with Hanks Balanced Salt Solution + HEPES containing calcitonin or PTH (0.01–1000 nM for dose-response or 10 nM for siRNA studies, both hormones from Tocris, Minneapolis, MN) for 15 min at 37 °C. Cells were then incubated with 70% ethanol at -20 °C for 3 h, the cells scraped, the mixture dried, the pellet re-suspended in water, and total cell cAMP determined by enzyme immunoassay (Enzo Life Sciences, Farmingdale, NY). Total cell protein was determined by the Bradford assay (Bio-Rad, Hercules, CA).

### RNA quantitation by real-time PCR

RNA was measured from both cell lines at baseline and after the relevant siRNA treatment. In addition, RNA was measured from DCT that were microdissected from C57BL6 mice, aged 2–3 months. The RNA was isolated using the RNeasy Mini Kit and reverse transcribed with Omniscript RT Kit (Qiagen, Valencia, CA). In addition, AC isoform and GAPDH mRNA levels were determined by real-time PCR (StepOne Plus, Applied Biosystems, Foster City, CA) using the Taqman Gene Expression Assay for AC1 (catalog # Mm01187829_m1), AC2 (Mm00467874_m1), AC3 (Mm00460371_m1), AC4 (Mm01323891_m1), AC5 (Mm00674122_m1), AC6 (Mm00475773_g1), AC7 (Mm00545780_m1), AC8 (Mm00507722_m1), AC9 (Mm00507743_m1) and GAPDH (Mm99999915_g1). The same amount of cDNA is used for each amplification and GAPDH values from each run are all within 0.5 cycles of one another.

### Determination of AC isoform mRNA expression

The presence of AC isoform mRNA was determined in 209 cells, D1 cells, and mouse brain (as a positive control). For mouse brain, frozen mouse tissue stored in the laboratory from past experiments was used; no mice were sacrificed for the purposes of these studies. Specific primers utilized and expected product sizes are shown in Table 1. PCR conditions for 30 cycles were 94 °C for 30 s, 58–62 °C (depending on the AC isoform) for 30 s, and 72 °C for 3 min, followed by electrophoresis through agarose.

**Table 1:** Primers used for adenylyl cyclase isoform mRNA detection (5′ to 3′)

	Forward primer	Reverse primer	Produce size (bp)
AC1	GCTGTTCGTGGTCACCAATGTCCG	GAGCGCTCTGTCAAGATCCGCACG	402
AC2	CAACACTGTCAACGTCGCTAGTAG	GAGCACGTACGTAATCAAGACGAAG	310
AC3	CCAGTCACTGGAGGTGAAGATGAA	GATGCTGACATTCTCGTGCCGGTA	179
AC4	CACCATGGTGGAATTTGCAGTGGC	GAGGATCTTCGAAGAGGGGAGCTC	387
AC5	CAATACAGTGAATGTGGCCAGCCG	CAGCAAAGGCAGAAGTTGCTTCTG	336
AC6	GGAAAGTAGATCCTCGCTTCG G	ACCGAGAAGATTCCAACCGC	262
AC7	GTACACTCCAGGCCATCTCG	TCCGCAGGAAAACAGAGCAT	261
AC8	GCATACAAGAGATCAACAAGCATTC	CTGGTCCTTCAGGATAAGGTAGGT	212
AC9	CATTGTGATGTCCCCCTTGGA	GTAGGGGATGATGTTCCGCA	283

### Statistics

Data are presented as mean ± standard error. Changes in cAMP responses after siRNA treatment were analyzed by the unpaired Student’s t-test. *P* < 0.05 was taken as significant.

## Results

### Characterization of DCT cell line hormonal responsiveness

**Fig. 1:**
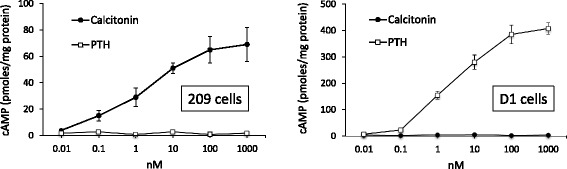
Dose response of calcitonin- and PTH-stimulated cAMP accumulation in distal convoluted tubule 209 and D1 cells. N = 3 each data point

The 209 and D1 cell lines are well characterized cell lines with respect to their retaining the in vivo DCT cell characteristics of Na^+^/Cl^−^ cotransporter expression and regulation, plasma membrane Ca^2+^ ATPase and Na^+^/Ca^2+^ exchanger-1 expression, as well as lack of thick ascending limb transporter activity [6, 8]. However, the expression and activity of the calcitonin and PTH receptors (two of the key hormones regulating DCT function) varies depending upon the subclone; D1 cells were developed to study the expression and regulation of PTH receptors that can be lost in the original 209 cell lines [7]. Consequently, the first set of studies were undertaken to determine PTH and calcitonin responsiveness, as assessed by cAMP accumulation in 209 and D1 cells. Exposure of 209 cells to varying concentrations of calcitonin dose-dependently increased cAMP accumulation (Fig. 1); in contrast, PTH up to 1 μM had no effect on 209 cell cAMP content. D1 cells dose-dependently increased cAMP levels in response to PTH, while calcitonin up to 1 μM had no effect (Fig. 1). Note that PTH triggered a cAMP signal that was ~5-fold greater than that elicited by calcitonin; the reasons for this are speculative and could be due to either the PTH receptor coupling more efficiently with Gs than the calcitonin receptor and/or that PTH receptors are expressed in higher numbers than calcitonin receptors. In addition, D1 cells stably transfected with the PTH receptor lost calcitonin signaling; the reasons for this are also unclear but may relate to cross-desensitization between PTH and calcitonin receptors [9]. Consequently, 209 cells were used to analyze calcitonin regulation of cAMP, whereas D1 cells were used to determined PTH modulation of cAMP.

### Determination of adenylyl cyclase isoform mRNA expression in DCT cell lines

**Fig. 2:**
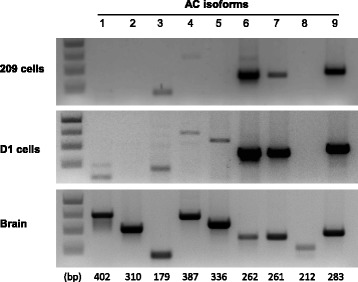
PCR of mRNA for adenylyl cyclases 1–9 in mouse distal convoluted tubule 209 and D1 cells. Mouse brain was used as a positive control for all 9 isoforms. Results are representative of 3 separate samples. Product sizes in base pairs (bp) are shown under each lane

The presence of AC isoform mRNA was initially assessed using non-quantitative PCR (Fig. 2). 209 cells expressed AC3, AC4 (albeit a very faint band), AC6, AC7 and AC9. D1 cells had an identical pattern of AC isoform expression with the exception that a faint band for AC5 was detected. No bands of the expected size for AC1, AC2 or AC8 were observed in either DCT cell line. Next, quantitative PCR for AC isoform mRNA was performed (Table 2); AC1, AC2, AC5 or AC8 were not observed in either DCT cell line. However, in both cell lines, AC6 was the most abundant AC isoform followed by AC9. AC3 and AC7 mRNA were variably expressed in the two DCT cell lines; AC4 was detected, albeit in relatively very small amounts. The PCR efficiencies for all 9 AC isoforms were tested using brain mRNA as a template; similar efficiencies were obtained for all 9 AC isoforms (data not shown).

**Table 2:** Levels of adenylyl cyclase isoform mRNA in distal tubule cell lines

	209 Cells		D1 Cells	
AC isoform	AC-GAPDH (ΔCT)	Relative abundance	AC-GAPDH (ΔCT)	Relative abundance
AC1	ND	0	ND	0
AC2	ND	0	ND	0
AC3	12.82 ± 0.09	50 ± 4	18.17 ± 0.9	1.5 ± 0.2
AC4	18.46 ± 1.11	1 ± 0.2	18.71 ± 1.4	1 ± 0.2
AC5	ND	0	ND	0
AC6	8.89 ± 0.06	760 ± 51	8.74 ± 0.2	1003 ± 93
AC7	17.02 ± 0.05	2.7 ± 0.3	13.84 ± 0.8	29 ± 3
AC8	ND	0	ND	0
AC9	10.67 ± 0.04	221 ± 19	10.21 ± 0.7	362 ± 30

*N* 3 each data point, *ND* Not detected

To compare the DCT cell lines with intact DCT AC mRNA expression, DCT were microdissected from mice and AC mRNA abundance determined (Table 3). Similar to the DCT cell lines, AC1 and 8 were absent, AC2 was in relatively low abundance and AC6 was the predominant AC isoform in microdissected DCT. Also similar to the DCT cell lines, AC3, AC7 and AC9 were present in acutely isolated DCT, albeit AC7 and AC9 were not in as great relative abundance compared to DCT cell lines when normalizing to AC4 mRNA levels. The biggest difference between DCT cell lines and acutely isolated DCT was that the latter contained AC5 mRNA.

**Table 3:** Levels of adenylyl cyclase isoform mRNA in acutely isolated distal convoluted tubules

AC isoform	AC-GAPDH (ΔCT)	Relative abundance
AC1	ND	0
AC2	12.80 ± 0.43	0.21 ± 0.01
AC3	10.32 ± 0.42	1.1 ± 0.02
AC4	10.51 ± 0.18	1 ± 0.01
AC5	10.07 ± 0.38	1.3 ± 0.01
AC6	5.94 ± 0.17	23.8 ± 1.2
AC7	10.44 ± 0.44	1 ± 0.01
AC8	ND	0
AC9	10.15 ± 0.29	1.3 ± 0.02

*N* 5 each data point. *ND* Not detected

### Effect of siRNA knockdown of AC isoforms on hormonal cAMP responsiveness

**Fig. 3:**
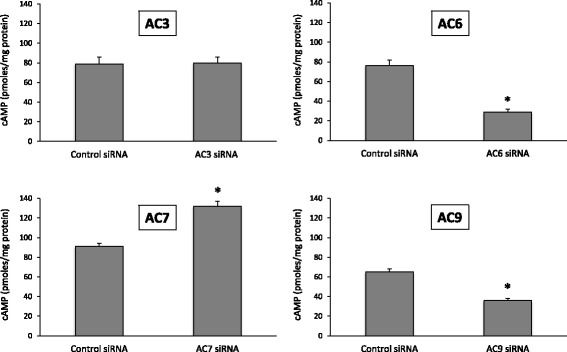
Effect of scrambled (control) or adenylyl cyclase isoform siRNA on calcitonin (10 nM) stimulated cAMP accumulation in distal convoluted tubule 209 cells. *N* = 9–12 each data point. *p < 0.05 vs. control

Based on the qualitative and quantitative mRNA expression findings, the effects of siRNA selective knockdown of AC3, AC6, AC7 and AC9 on hormonal cAMP responsiveness were determined. Isotype-specific siRNA was utilized since pharmacologic agents are not available that specifically target each individual AC isoform. In 209 cells, optimization of siRNA silencing conditions resulted in a maximal decrease of AC isoform mRNA levels as follows: AC3 = 35 ± 4% of control, AC6 = 38 ± 6% of control, AC7 = 38 ± 5% of control, and AC9 = 39 ± 6% of control, *N* = 4 each data point. Treatment with siRNA only reduced the targeted siRNA species in 209 cells, i.e., AC3, AC6, AC7 and AC9 mRNA levels were not reduced by siRNA that did not specifically target them (data not shown). In the absence of calcitonin, 209 cells treated with vehicle, scrambled siRNA, or AC isoform-specific siRNA had no detectable cAMP. We next examined the effects of siRNA on calcitonin-stimulated cAMP. 10 nM calcitonin was used for these studies since this concentration of calcitonin elicited ~50–60% of maximal cAMP accumulation in the dose-response studies. Treatment with AC6 and AC9 siRNA reduced calcitonin-stimulated cAMP accumulation by roughly comparable degrees (45–60%) (Fig. 3). AC3 siRNA had no effect. Surprisingly, AC7 siRNA augmented calcitonin-induced cAMP levels (by ~30%).

**Fig. 4:**
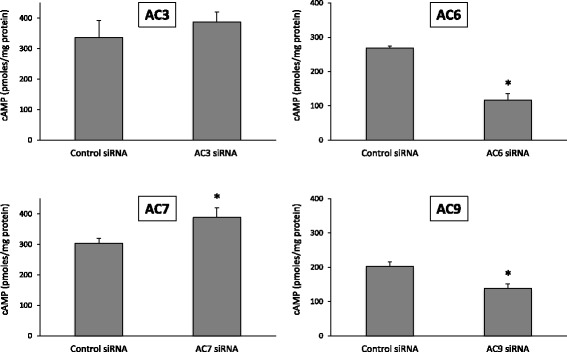
Effect of scrambled (control) or adenylyl cyclase isoform siRNA on PTH (10 nM) stimulated cAMP accumulation in distal convoluted tubule D1 cells. *N* = 9–12 each data point. *p < 0.05 vs. control

Similar siRNA knockdown studies were conducted on PTH effects in D1 cells. Optimization of siRNA conditions resulted in a maximal decrease of AC isoform mRNA levels in D1 cells as follows: AC3 = 35 ± 7% of control, AC6 = 39 ± 4% of control, AC7 = 50 ± 11% of control, and AC9 = 29 ± 3% of control, *N* = 4 each data point. Treatment with siRNA only reduced the targeted siRNA species in D1 cells, i.e., AC3, AC6, AC7 and AC9 mRNA levels were not reduced by siRNA that did not specifically target them (data not shown). In the absence of PTH, D1 cells treated with vehicle, scrambled siRNA, or AC isoform-specific siRNA had barely detectable cAMP levels (2–3 pmoles cAMP/mg total cell protein). AC6 and AC9 siRNA reduced PTH-stimulated cAMP accumulation; AC6 siRNA reduced cAMP levels by 60%, while AC9 siRNA elicited a 30% decrease (Fig. 4). Again, treatment with AC3 siRNA had no effect. As in 209 cells, AC7 siRNA increased PTH-induced cAMP levels (by ~20%) in D1 cells; notably, this effect was seen despite only being able to achieve 50% reduction in AC7 mRNA levels.

## Discussion

The current study identified AC3, AC6, AC7 and AC9 as the predominant AC mRNA isoforms in DCT cells. Importantly, this pattern of AC mRNA isoform expression was very similar between 209 and D1 cells. In addition, microdissected mouse DCT expressed similar AC isoform mRNAs as cultured DCT cells with the exception that AC5 was present in microdissected, but not cultured, DCT. Notably, AC3 has been localized to mouse DCT using immunostaining [10], whereas AC6 [11, 12] and AC9 [11] were detected in microdissected rat DCT. Microarray analysis of isolated mouse DCT has reported only AC6 [13] while serial analysis of gene expression libraries created from mouse nephron segments detected only AC6 and AC9 in the DCT [14]. Microarray analysis of rat DCT detected only AC5 and AC6 [15]. These transcriptomic studies, however, all have thresholds of detection that can miss rare mRNA species, hence it is not possible to rule out expression of other AC isoforms. AC4 mRNA expression in DCT is unclear as it was detected by one group [11] but not by another [12]. In general, AC1, AC2 and AC8 mRNA have not been detected in DCT although one study reported AC2 mRNA in microdissected rat DCT [11]. One caveat is that almost all studies, including ours, did not assess AC isoform protein levels due to the very low abundance of these proteins coupled with limited tissue amounts. Thus, 209 and D1 DCT cell lines express AC isoforms, at least at the mRNA level, in a relatively similar pattern to that observed in vivo.

As mentioned in the Introduction, there are no studies that have specifically examined the role of individual AC isoforms in mediating hormonal actions on DCT cells. That said, whole animal AC6 knockout mice have reduced vasopressin-stimulated phosphorylation of NCC on threonine-58 [16]; since NCC is selectively expressed in the DCT, this raises the possibility that AC6 partly mediates vasopressin-stimulated cAMP-dependent NCC modulation in the DCT. Using the same global AC6 knockout mice, Fenton et al. [17] reported that serum calcium concentration was unaffected by AC6 deletion, however urinary calcium excretion was not assessed. Most importantly, bolus PTH increased urinary cAMP in control, but not AC6 KO mice, while blood PTH levels were markedly elevated in AC6 KO mice. In another study, mice with whole nephron AC3 knockout exhibited urinary Mg^2+^ wasting and hypomagnesemia on a low Mg^2+^ diet [18]. Because the DCT is a key site of Mg^2+^ reabsorption in the nephron, these studies suggest that AC3 in the DCT modulates Mg^2+^ handling. However, mRNA levels of TRPM6, the key apical membrane channel mediating Mg^2+^ reabsorption in the DCT, were elevated in the nephron of AC3 KO mice [18].

The current siRNA studies demonstrate that AC6 and AC9 are involved in mediating calcitonin and PTH stimulation of cAMP accumulation in cultured DCT cells, while AC7 activation may paradoxically inhibit hormone-stimulated cultured DCT cAMP accumulation. It is notable that a significant effect on hormone-stimulated cAMP of reducing siRNA to 35–40% of control values was observed (it is not possible, as described above, to measure AC isoform protein levels), indicating that even incomplete targeting of the AC isoforms achieves a clear biologic effect. These observations are important because they provide insights into signaling systems that modulate the effects of calcitonin and PTH on DCT cells - each AC isoform is uniquely regulated by G proteins, divalent cations, small molecules, post translational modification, and subcellular localization [19]. Adenylyl cyclase 6 is activated by Raf1 and inhibited by nitric oxide, protein kinase C (PKC), protein kinase A (PKA), low intracellular concentrations of Ca^2+^, and Gα_i_ [19, 20]. By contrast, AC9 is the only AC isoform inhibited by calcineurin, which, like AC6, it is inhibited by PKC and Gα_i/o_, and activated by Gα_s_ and Gα_q_ [19]. Of note, AC6 appears to be restricted to lipid rafts [21]; the subcellular localization of AC9 is not clear. Such subcellular localization is potentially important because cAMP is found in microdomains within cells and is closely associated therein with phosphodiesterases, PKA, A-kinase anchoring proteins, and gravin [22]. In contrast to AC6 and AC9, AC7 is activated by PKC and stimulated by Gα_i_ [19, 23]. Thus, calcitonin and PTH regulation of AC6, AC7 and AC9 activities may involve pathways that uniquely activate AC6 and AC9 to promote cAMP accumulation, and other pathways that individually activate AC7 to limit the magnitude of the cAMP increase.

We did not observe an effect of siRNA knockdown of AC3 on PTH- or calcitonin-stimulated cAMP. This could be due to incomplete targeting of AC3 in the DCT cells. However, a recent study by Blanchard et al. [18] reported that mice with nephron-specific AC3 knockout did not have altered urinary calcium excretion as compared to control mice. While PTH and calcitonin levels were not assessed in these mice, the normal calcium excretion suggests that nephron AC3 in general, and DCT AC3 in particular, may not be essential for hormonal regulation of renal calcium handling.

The present study did not specifically explore the intracellular signaling pathways that are involved in PTH and/or calcitonin regulation of DCT AC isoform activity; such studies are important but were beyond the scope of these initial observations. Another key finding that needs further clarification is how AC7 knockdown increases hormonal-stimulated cAMP levels in DCT cells. We could not find examples in the literature of an interplay between AC isoforms wherein activation of one isoform reduced the increase in cAMP content catalyzed by other isoforms. As noted above, AC-stimulated cAMP accumulation occurs in discrete cellular microdomains, so it is conceivable that activation of a given AC isoform could modulate the activity of another through locally induced signaling molecules. While the nature of such pathways is entirely speculative, one possibility is that AC7 might activate a cAMP-dependent phosphodiesterase that reduces AC6 and/or AC9 induced cAMP accumulation. Clearly, this represents an important area in need of further study. Finally, these studies were conducted in DCT cell lines; conclusions about the in vivo role of DCT cell AC isoforms cannot be directly drawn. Unfortunately, there is no way to specifically target the entire DCT in vivo, hence such studies will await future technological developments.

## Conclusions

This study identifies the specific AC isoforms mediating the cAMP-stimulating effects of calcitonin and PTH in cultured DCT cells. We report that AC6 and AC9 mediate the stimulatory effects of calcitonin and PTH on cAMP accumulation, while AC7 paradoxically inhibits hormone-stimulated cAMP content accumulation. These studies are important for at least 3 reasons: 1) since many of the cell signaling pathways have been described by which specific AC isoforms are regulated, the current studies, by identifying which of these AC isoforms mediate hormonal action in at least cultured DCT, will facilitate future studies looking at how these hormones exert their biological effects; 2) these studies have uncovered an unusual inhibitory effect of AC7 on hormone-stimulated cAMP accumulation; and 3) these studies will potentially identify a possible role for specific AC isoform agonists and/or antagonists in modulating electrolyte handling by the distal tubule; such agents are in active development.
